# Care Practices Supporting Older Adult Mobility in Institutional Long‐Term Care: An Ethnographic Study

**DOI:** 10.1111/scs.70308

**Published:** 2026-08-19

**Authors:** Johanna Finskas, Minna Stolt, Riitta Suhonen, Noora Narsakka

**Affiliations:** ^1^ Department of Nursing Science University of Turku Turku Finland; ^2^ Wellbeing Services County of Satakunta Pori Finland; ^3^ Turku University Hospital, Wellbeing Services County of Southwest Finland Turku Finland

**Keywords:** ethnographic study, institutional long‐term care, mobility, mobility care, nursing care, older adults

## Abstract

**Background:**

Older adults living in institutional long‐term care (ILTC) generally have decreased mobility, and their mobility care is often insufficient. Residents spend much of their day being sedentary, even though sedentary behaviour and inactivity are known to have many negative effects on health and functional capacity. More qualitative research describing care practices that support residents' everyday mobility in ILTC is needed. The aim of this study is to describe how care workers support residents' mobility in one ILTC unit.

**Methods:**

A focused ethnographic study was conducted. Data were collected by observing care workers (*n* = 10) and residents (*n* = 12) in one ILTC unit in May and June 2023. Data collection was guided by Spradley's framework. In addition, informal interviews were conducted with the care workers during the observations. The data were analyzed using thematic analysis.

**Results:**

Care workers carried out mobility care through situational compromises between person‐centred mobility care and institutional expectations. While mobility care was person‐centred, supporting residents' autonomy and grounded in residents' mobility needs, it was also shaped by institutional expectations. Institutional expectations were evident when other essential care activities were prioritized and routine practices were utilized in mobility care.

**Conclusions:**

Mobility care occurs in the interaction process between care workers and residents. Supporting residents' mobility requires care workers to balance person‐centred mobility care and institutional expectations. Therefore, care workers need guidance for communication with residents with dementia, sufficient resources, and shared practices to enable quality mobility care in ILTC. Mobility care should also be embedded in institutional values and guidelines to prevent it from becoming a mechanical activity of the residents' day.

## 1 Introduction

It is essential that care practices and physical environment in institutional long‐term care (ILTC) support older adults' ability to be mobile [1, 2] because mobility is a basic human need [3]. Mobility has several positive impacts on older adults' physical functioning [4], independence, and quality of life [5]. In addition, older adults themselves have reported that mobility signifies independence and freedom, which positively affects not only their physical function but also their social and mental health [6]. Therefore, older adults in ILTC should have opportunities to improve, optimize, and maintain their mobility. Despite this, previous research has shown that older adults living in ILTC spend most of their day sedentary, in sitting or lying positions [7, 8]. This may lead to immobility and mobility limitations, contributing to lower physical activity levels and increasing the risk of falls and social isolation [9, 10]. Mobility care is recognized as a critically unmet care need, which is often insufficient or completely ignored in ILTC settings [11, 12].

A major cause of dependence and disability among older adults is memory disorder. It is also the most common reason ILTC services are needed when disorders progress in later stages and become associated with high levels of dependency and complex care needs [13, 14]. Most professionals in ILTC settings are care workers such as nurses [15]. They provide most of the direct care to older adults, which should include meeting their need for movement [16, 17], also referred to as mobility care. Therefore, care workers in ILTC need competence in providing person‐centred care that supports individual needs and functional capacity in older adults with dementia [13]. Key elements in person‐centred care are respecting older adults' values, needs, and preferences, which should guide all aspects of care in ILTC. These should be visible in an individualized, goal‐oriented care plan that is regularly reviewed to ensure that care practices are adapted to each individual's personality, health status, and circumstances [18]. However, in relation to mobility care, it has been noted that care workers may be unaware of it or may not recognize it as their responsibility; some care workers may consider the mobilization of older adults as primarily belonging to the role of a physiotherapist [19].

This study examines the movement of older adults through the concept of *mobility*, as many older adults in ILTC, also referred to as residents, are dependent on some form of assistance to be able to move from one place to another. Mobility refers to an individual's ability to move and to be moved freely [20]. It can be seen as a continuum, ranging from no impairment to completely immobile [21] and can include using a mobility aid and the assistance of others [22].

Previous studies in ILTC settings have focused on mobility mostly in terms of physical rehabilitation, delivered by research staff or a physiotherapist [23]. The effects of various interventions to support mobility [17], such as walking interventions [24, 25, 26], sit‐to‐stand interventions [27, 28], and function‐focused care interventions [29, 30] have been examined. In these interventions, residents' individuality and personal goals have been an integral part of supporting their mobility [26, 30]. However, the importance of supporting mobility as part of routine care practices has also been recognized [31]. There is a need to understand current mobility care practices and provide qualitative descriptions of *how* care workers support older adults' mobility, especially when memory disorders may hinder the ability to receive, remember, or follow instructions. To understand the care routines or tacit knowledge care workers use when resolving complex and sudden situations in everyday care, ethnographic research has been utilized [32, 33]. Participant observation in ILTC can reveal the tacit, situational, and everyday interactions [33] in mobility care situations that may occur routinely without conscious intention. The aim of the study is to describe, through ethnographic observations and informal interviews, how care workers support the mobility of older adults in ILTC. This knowledge is valuable in the development of older adult care culture. The study was guided by one main research question: How do care workers support residents' mobility in one ILTC unit?

## 2 Materials and Methods

### 2.1 Study Design

Focused ethnography involving observations and informal interviews was conducted in one ILTC unit in Finland in 2023. The aim of the study justified the choice of focused ethnography, which provided an opportunity to observe a specific topic among a defined target group, over a limited time frame, and in a natural care environment [34, 35]. Due to the ILTC context, in which older adults are dependent on care workers' support for mobility, mobility care occurs primarily through social interaction. Therefore, the ethnographic approach was guided by symbolic interactionism, a sociological perspective that focuses on how individuals construct meaning through social interaction, emphasizing the role of culture and social encounters between care workers and residents [36, 37]. Symbolic interactionism presumes that people act based on the meanings things have for them. These meanings arise from social interaction with others and are continuously interpreted and modified through an ongoing interpretive process [37]. This theoretical approach directs attention to how care workers interpret mobility‐related circumstances in close interaction with residents and how they decide their course of action when providing mobility care [38].

### 2.2 Study Setting

At the time of data collection, in 2023 in Finland, the average age of older adults living in ILTC was 84 years [39]. ILTC is round‐the‐clock care provided for older adults who require continuous care and whose care cannot be appropriately arranged through home care, informal care, family care, or other services [40]. According to Finnish legislation, ILTC should be provided to ensure residents' safety and to maintain their social interaction and engagement in meaningful activities that support well‐being and functional capacity [41]. Most ILTC staff are care workers, and required staffing levels are defined in legislation to ensure the quality of care [41]. Rehabilitation workers, such as physiotherapists, occupational therapists, and social workers, account for only 2% of the total staff [42].

One ILCT unit was recruited through an open call as part of the larger participatory action research project, ‘Increasing older adults' physical activity in ILTC’ [31, 43]. The unit, home to 25 residents, served as the setting for this ethnographic research. The unit consisted of two long hallways with residents' rooms and common areas, including small kitchens, living rooms, a sauna, and a balcony at each end. Each resident had a private room and bathroom. According to national staffing requirements, five to six care workers were present during daytime, each assigned to care for four to five residents. Care workers had no separate break rooms allowing them to remain close to the residents during their shift.

### 2.3 Participants

In the larger participatory action research project [31, 43], all staff, residents, and family members from the recruited unit were invited to participate. Eligibility required the capacity to give informed consent in Finnish. This ethnographic study included a sub‐sample of 10 care workers and 12 residents (Table 1) from the larger project who were present in the unit during data collection. No participants withdrew during the study.

**TABLE 1:** Participants' background information.

	Residents (*n* = 12)	Care workers (*n* = 10)
Women, %	58	100
Age, range	65–102	21–66
Age, mean	80	43
**Assistance needed in mobility**		
No assistance^a^, %	25	
Occasionally assistance^b^, %	33	
Full assistance^c^, %	42	

^a^ Can walk without guidance of care worker with or without assistive device.

^b^ Needs assistance transferring to wheelchair, in wheelchair can be mobile independently or can walk mainly without guidance, but occasionally needs assistance from care worker.

^c^ Needs full assistance and presence of care worker to be mobile.

### 2.4 Data Collection

The first author, JF, carried out ethnographic fieldwork, with focused observations and informal interviews, in May and June 2023. Observation time totaled 35 h and included 60 mobility care situations. Spradley's (1980) framework guided the data collection [36] (Table 2). Observed situations included daily routines and focused on care worker–resident interactions that supported residents' mobility. Observations took place in common areas and residents' rooms several times a week, 1–3 h at a time, between 8:00 a.m. and 8:00 p.m. Observed situations varied from residents' daily care activities, such as eating and hygiene care, to other activities, such as taking out trash or using an exercise bike. The situations were initiated by either residents or care workers.

**TABLE 2:** Spradley's framework for guiding data collection.

Spradley's framework	How has it been applied in relation to the research topic?	An example of an observed mobility situation
Space	Where does the observed mobility situation happen?	Dining room in the unit
Actor	Who is involved in the situation?	One care worker, one resident
Activity	What kind of activity happens in a mobility situation?	The resident is sitting at the table and is trying to stand up but cannot do it alone because the table is in the way.
Object	What kind of objects, accessories, or devices are involved in the situation?	Dining table and chairs
Event	In what events do care workers support mobility?	Before dinner, waiting for dinner to come, residents' leisure time
Act	What are the different acts that happen in the situation?	The resident is trying to stand up but cannot do it alone. The care worker walks by and notices this. The care worker moves the chair away from the table by dragging the chair along the floor and creates space for the resident to stand up. The resident stands up right away and starts walking. The care worker continues their way.
Time	When is a situation observed? What is the duration?	About 4 p.m., the situation lasts about a couple of minutes.
Goal	What is the goal of the situation? At whose initiative?	The care worker's goal is to help the resident to walk by giving enough space. The resident's initiative.
Feeling	Are there emotions seen in the situation?	The resident is clearly frustrated because they cannot get up from the chair. The resident makes sounds without clear words, looks around, taps the table, and tries to push the table away with their hands. The care worker is in a hurry and walks past quickly, but when they notice the situation, they help the resident in a cheerful way and then continue to another resident.

To gain a deeper understanding of participant perspectives and clarify observations, informal interviews were conducted during observations. Interviews were situational and were held when time and environment allowed. For example, interviews were not conducted during restless situations or when care workers were on their way to other residents. Conversations began with the researcher summarizing an observed situation. Care workers were asked open‐ended questions to encourage free interaction [36], prompting them to describe, reflect, and clarify observed situations and their actions. Ten short informal interviews were conducted. Observation and interview data were considered a single dataset, recorded pseudonymously as handwritten field notes and elaborated upon digitally immediately afterwards. Video recording was not used, as it can affect participant behaviour [44]. Data collection was completed when mobility care situations became repetitive, no new mobility‐support strategies emerged, and the phenomenon could be adequately described in writing [44].

### 2.5 Reflexivity

The first author (JF) had a dual role. In addition to conducting research, the first author had been working as an occupational therapist in the participating unit 1 day per week for 2 years. This suited the focused ethnographic approach, as the researcher's role was inherently participatory [45]. This also allowed data collection to start more quickly and shortened the ethnographic familiarization phase. However, reflexivity was carefully taken into consideration. Before entering the field, the first author's dual role was discussed within research team and considered through SWOT analysis. Because of this dual role, a risk of misinterpretation and preconceived assumptions was identified, as well as a potential sense of pressure for participants to take part due to the researcher's familiarity. These were discussed within the unit and with participants during and after data collection and by intentionally approaching each situation with an open mind as a listener and learner [46]. The researcher's role was explicitly communicated to the participants with a name tag, a university t‐shirt, calendars, and in staff meetings. The first author kept a reflexive diary throughout the data collection process to critically examine personal assumptions and their position in the field.

All members of the academic research team have practice‐based bachelor's degrees in healthcare and work experience in ILTC. MS and RS have extensive experience in research on older adults. NN is a postdoc researcher, while JF is a recently graduated MHS. All researchers are white women residing in Finland.

### 2.6 Data Analysis

The data was analyzed (JF) using thematic analysis [47], a flexible and data‐driven approach that allows full use of data without excluding any information. Quantifying or conceptualizing the data was unnecessary, which also supported the choice of thematic analysis [47]. The analysis was conducted over six phases: familiarization with the data, coding, theme‐searching, theme‐reviewing, theme‐naming, and reporting [47]. Data analysis began from the first moments in the field and continued until a sufficient understanding of the phenomenon was developed [48].

There were 30 pages of written data (Times New Roman, font size 12, line spacing 1.5). During the familiarization and coding phases, the data was read multiple times and coded (JF) inductively using NVivo software version 12, guided by the study's research question (Table 3). In the theme‐searching phase, the codes were examined, compared, and combined according to similarities and consistencies. In theme‐reviewing phase, the grouped codes were reviewed and refined into sub‐themes and main themes to reflect the story of the data. Themes were then named and described in writing, and finally reported [47]. In theme‐reviewing, naming and reporting phases the sub‐themes and main themes were discussed with another researcher (NN). The data was revisited frequently throughout the process to ensure that the themes reflected the data [47].

**TABLE 3:** An example of the data analysis process.

Field notes	Code	Subtheme	Main theme
*The care worker explains that it is important to let the resident drink milk on their own, if they are able to do it. This supports the residents' own habits and allows own movement*.	Allow own movement Support the resident's own habits	Supporting residents' autonomy in mobility care	Providing person‐centred mobility care
*The researcher met the resident and the care worker at the unit's outdoor area. The care worker explains they were going to do stair walking. – Afterward, the care worker explained that stair walking is a planned activity to support the resident's walking ability*.	Planned activity Support walking ability	Grounding mobility care in residents' mobility needs	
*The resident and the care worker walk hand in hand toward the balcony. Near the balcony door, the care worker goes ahead and opens the door so the resident can enter*.	Walk hand in hand Opens the door for the resident Expanding one's life space		

### 2.7 Ethical Considerations

The study followed the European Code of Conduct for Research Integrity [49]. Ethical approval from the University Ethics Committee (TY/40/12.12.2022, TY/131/06.01.01/2024, TY/699/06.01.01/2023) and a research permit from the recruited organization (2023‐001224T 13 02 01) were obtained as a part of the larger research project. Written informed consent from participants was obtained individually by NN, who informed all participants of the purpose of the study, its voluntary nature, and potential harms and benefits. The study involved participants with memory disorders, and their family members provided consent to participate if the care workers evaluated them as being unable to provide consent themselves. Informed consent was continuously assessed throughout the research process, including oral confirmation of participants' willingness to participate before observations. The study is reported following the Consolidated Criteria for Reporting Qualitative Research (COREQ checklist) [50]. Microsoft Copilot was used to support language editing by improving individual sentence structures and clarity. The authors take full responsibility for the integrity, accuracy and content of the work.

## 3 Results

The analysis identified one overarching theme comprising two main themes and four sub‐themes, describing care workers' support for residents' mobility in ILTC. The overarching theme illustrates how mobility care was provided through situational compromises with two distinct main themes: person‐centred mobility care and institutional expectations (Table 4).

**TABLE 4:** Supporting resident’ mobility in ILTC.

Overarching theme	Main theme	Sub‐theme
Mobility care is carried out through situational compromises	Providing person‐centred mobility care	Supporting residents' autonomy in mobility care
		Grounding mobility care in residents' mobility needs
	Institutional expectations shape mobility care	Prioritizing other essential care needs
		Utilizing routine practices in mobility care

### 3.1 Mobility Care Is Carried Out Through Situational Compromises

Mobility care was provided through situational compromises made by care workers, reflecting how they interpreted and negotiated meanings of mobility care in ongoing interaction with residents. While mobility care was person‐centred, it was also the result of institutional expectations that shaped its implementation. The culture observed in the unit was largely mobility supportive; for example, mobility was not directly restricted. It was evident that care workers respected residents' autonomy and residents' needs were considered in mobility care. However, there were situations where care workers reinterpreted priorities resulting in the prioritization of other essential care needs and the utilization of routine practices in mobility care. These illustrate how mobility care is continuously constructed through ongoing interpretive processes [37], in which care workers balance competing meanings between residents' preferences and organizational expectations.

#### 3.1.1 Providing Person‐Centred Mobility Care

Person‐centred care was one of the major factors shaping the unit's atmosphere and assumptions regarding residents' mobility. This was evident, for example, when residents were listened to or approached warmly in interaction during mobility care. In addition, care workers utilized flexible working manners and situational awareness reflecting how meanings of mobility care are formed and evolved within resident and care worker interaction. Person‐centred mobility care appeared to comprise supporting residents' autonomy and grounding mobility care in residents' needs.

Due to cognitive limitations, some residents could not express themselves fluently or clearly during mobility care, which automatically placed care workers in a dominant role. Nevertheless, care workers recognized the meaning of mobility from the residents' perspective by consciously adapting their approach to support residents' autonomy, for example, by allowing time to perform an activity, motivating, and offering choices regarding mobility. This highlights the delicate balance between residents' progressively declining physical functioning and care workers' commitment to supporting autonomy. From the care workers' perspective, supporting residents' autonomy and decision‐making sometimes meant accepting behaviour that appeared illogical, such as residents moving on hands and knees or choosing to sleep on a mattress on the floor rather than a bed. This shows how interpretation is not fixed on established meanings but is a flexible process in which meanings are continuously used and revised to guide care workers' actions [37]. For example, residents' seemingly illogical behaviour is interpreted in ways that support their autonomy.

In the interviews, care workers explained the meaning of mobility for residents' autonomy from practical, person‐centred perspectives, recognizing even small or unconventional movements that positively affected residents' well‐being and physiological functioning. In addition, by supporting residents' autonomy and allowing self‐initiated movement, care workers reported that residents' own habits were maintained, and they were calmer and more satisfied during the day.

The care worker asks the resident to stand. The resident does not respond and looks at the floor. The care worker waits quietly and asks again, but the resident still does not move. The care worker then calls the resident by name. This time, the resident looks up and suddenly stands. Afterwards, the care worker explains that it is best to give this resident time and gentle encouragement, and to avoid holding their hands, since the resident prefers to act independently and at their own pace. (Field note, resident room, morning shift)

It was observed that person‐centred mobility care was grounded in residents*'* mobility needs. These needs include changing functional ability, moving freely, and expanding the living space. To meet these needs and provide person‐centred mobility care, care workers constructed and guided their actions by interpreting the meaning of residents*'* actions during mobility care situations. This adaptability was evident when care workers switched between assistive devices or modified guidance approaches to better respond to a resident*'*s need to walk or a changed functional ability. Need‐based mobility care also included planned mobility activities used to systematically maintain individual functional ability.

The care worker approaches the resident, calls them by name and invites them to do hand exercise using the exercise bike. They walk together to the living room. The resident does not make eye contact and speaks quietly, repeating a few syllables. The care worker guides the resident to a chair and asks them to sit several times. After a while, the resident sits down independently and the care worker moves the chair in front of the exercise bike. The resident takes hold of the hand pedals and begins pedaling independently. The care worker then puts on music for the resident. (Field note, hallway and living room, evening shift)

Residents' need and desire to move alone without help was respected. In some situations, residents' functional ability was clearly reduced, and walking alone was unsteady. Still, care workers let residents walk alone and freely back and forth in the hallway. One care worker described prioritizing the residents' willingness to walk over concerns about the risk of falling. This reflects how the meaning of mobility was negotiated in interaction with the residents, leading care workers to prioritize autonomy as the guiding meaning rather than adopting a risk‐related meaning.

The care worker talks about a resident whose mobility was worse when they first arrived at ILTC. The residents walking with a rollator was difficult and unsteady. Over time, the resident expressed a wish to walk and gradually began to walk more. Care workers allowed the resident to walk independently and the resident's walking ability improved. The care worker emphasizes that sometimes there is no need to be afraid of residents falling, but instead to let residents walk on their own and see this in a positive way. (Field note, resident room, morning shift)

Residents' need to expand their living space at the ILTC facility was supported. Although the care workers could not change the built environment of the unit, they used practical awareness to enable and facilitate the residents' need to move within it. This simply meant that care workers fit their actions in line with the actions of residents [37]. These actions included small adjustments taken by the care workers, such as opening the door of a balcony or a resident's room, moving a chair out of the way, rearranging furniture to create more space, and moving objects closer to the residents' reach.

#### 3.1.2 Institutional Expectations Shape Mobility Care

Several institutional expectations affecting mobility care were observed. ILTC settings were seen as establishing social structures that positioned care workers as carers and residents as recipients of care, contributing to the prioritization of other essential care needs over mobility care. Also, routine practices as a part of institutional expectations shaped the way mobility care was provided, with mobility sometimes primarily valued for its role in enabling daily care. Residents' basic needs, such as hygiene and nutrition, were seen as major goals, and mobility care was considered the method of ensuring that these needs were met. For example, care workers helped residents get up from bed at lunchtime or assisted them in walking when there was need to visit the toilet. Negotiation and situational compromises were evident as other essential care needs sometimes took precedence over residents' immediate need to move.

The care worker enters the resident's room and tells the researcher that they will help the resident with evening care such as washing and changing into nightwear. The resident is lying in bed, and because of hemiplegia, cannot get out of bed without assistance. The resident greets the care worker cheerfully and asks for assistance in getting into a wheelchair. However, the care worker replies that they will do the evening care first and help the resident change into nightwear before moving them to the chair. The resident accepts this order. (Field note, resident's room, evening shift)

There were also situations when a resident resisted getting up from bed or going to the toilet, but institutional expectations regarding limited time resources and pre‐scheduled mealtimes meant that it was necessary to do so at that time. Care workers also described the meaning of mobility care from a carer's perspective in informal interviews. They emphasized how supporting mobility enabled residents to eat independently or made care less physically demanding for care workers. This can be understood as care workers being objects of their own actions, meaning that their actions are also guided by how they define themselves and how they orient their actions toward residents [37]. Due to care workers' task‐oriented approach and institutional expectations, residents were observed leaving mobility‐related decisions to care workers.

The resident sits in an armchair in the living room watching TV. The care worker approaches the resident and says it is dinnertime and the meal is ready. The care worker asks whether the resident wants to stay in the living room or move to the table with others. The resident looks at the care worker, laughs softly, and says it does not matter where they eat dinner. The care worker suggests going to the table and then assist the resident there using a returner transfer aid. (Field note, living room, evening shift)

Institutional expectations were seen to include routine practices in mobility care. These were often used to ensure safety or working ergonomics but were sometimes utilized without residents' consent. Assistive devices were used to enable residents' movement, while also used routinely to support care workers' ergonomics. Residents sometimes did not want to use assistive devices, forgot to use them, and therefore required care workers' support to utilize them which indicates how the same objects can have different meanings for different people [37], especially for residents with dementia. In addition, care workers routinely worked collaboratively, for example when using patient lifts. While collaboration among care workers was necessary, it reduced the space for residents' own voice, which tended to receive less attention in these situations.

As a part of institutional expectation, ILTC followed routine safety practices regarding the physical environment. Doors leading outside were kept locked, which was considered normal and self‐evident, but also limited residents' possibility to go outside. This specific ‘world’ for residents with memory disorder referring to a setting, surroundings and textures as understood in symbolic interactionism [37], was observed as lacking a symbolic meaning of home, making it unfamiliar and increasing residents' dependence on care workers.

The resident walks down the hallway toward the care worker and asks for help finding their own room and coat. The care worker interrupts what they were doing, smiles at the resident and says they will go together to find the room. Taking the resident's hand, they walk side by side down the hallway to the resident's room. (Field note, hallway, evening shift)

## 4 Discussion

This study revealed that care workers supported residents' mobility in one ILTC unit through situational compromises highlighting that mobility care is constructed through ongoing interpretation and interaction with residents. Care workers continuously interpreted residents' actions and constructed meanings that guided mobility care [37]. They provided person‐centred mobility care supporting residents' autonomy and grounding its implementation in residents' needs. However, institutional expectations, such as completing other essential care needs and utilizing routine practices, affected mobility care.

Situational compromises in mobility care reflected ongoing interpretive process of care workers, through which they negotiated meanings in specific situations [37]. These were evident when care workers tried to balance the needs of residents and the expectations of the institution. This balancing process has been widely recognized as shaping care workers' daily practices and care‐related decision‐making. Within ILTC, the organizational care system may set assumptions that lead to care workers following predetermined care needs defined by the organization rather than the residents' themselves [51]. Consequently, care prioritization is influenced not only by residents' needs but also by the demands of effective workflow [52]. At the same time, care workers experience practical constraints such as time pressure and multiple responsibilities, limiting opportunities for close interaction with residents [53]. Limited time resources and staff shortages have been identified as barriers to physical activity intervention implementation [17]. From the perspective of care workers, providing mobility care in this balancing process can be demanding. Therefore, it is crucial to increase the shared understanding of mobility care and reflect its meaning within organizational values that guide care. In addition, overcoming barriers in mobility care requires open communication, sufficient time resources, management support, and shared practices which together support the co‐construction of shared meanings of mobility care in ILTC.

Despite situational compromises, care workers carried out person‐centred mobility care where resident autonomy was supported, and mobility care was grounded in residents' needs. This is in line with previous research and guidelines suggesting that residents' individuality is an integral part of mobility interventions [26, 30] and that care provided in ILTC should be timely and sufficient in relation to individual needs [41]. When person‐centredness extends to everyday care practices, it may have crucial role in supporting residents' holistic well‐being. Mobility care may be conceptualized as an integral component of person‐centred care that enhances not only physical but also psychological and social dimensions of health [6]. From a physical perspective, encouraging residents to actively participate in movement and daily activities can help maintain functional capacity, muscle strength, and balance, reducing the risk of decline [4]. At the same time, mobility‐related interactions offer opportunities to support psychological well‐being by fostering a sense of autonomy, competence, and self‐efficacy [54]. When residents are enabled to move at their own pace and make choices about their movements, they are more likely to experience dignity and control in their daily lives [55]. These experiences can be understood as emerging through interaction, as residents construct meanings related to mobility, self and capability in relation to care workers [37]. Furthermore, mobility care can significantly contribute to social engagement and meaningful participation in social activities [43]. Future research could further explore residents' perspectives on the benefits and meanings they attribute to mobility.

Given the benefits of person‐centred mobility care, care workers' communication competence with residents with dementia is crucial for responding to residents' mobility‐related preferences and wishes. Residents did not always initiate contact with care workers or express themselves verbally, which limited clear verbal interaction. To utilize non‐verbal communication strategies such as haptics, kinesics, proxemics, or vocalics, care workers should be self‐aware and intentionally modify their communication style [56]. Also, symbolic‐level communication such as using old photos or music [57], as well as touch, can strengthen the connection to people with dementia [58]. In mobility care situations, care workers and residents continuously interpret each other's actions and form meanings related to movement and themselves [37], which makes social interaction a fundamental part of mobility care. Therefore, communication strategies should be included in mobility care education and workplace orientation as integral part as the use of assistive devices and ergonomic practices.

Mobility care was shaped by institutional expectations, where completing other essential care needs was given higher priority and routine practices were utilized. As a living environment, ILTC is characterized by implicit assumptions that position residents as care recipients in need of help and care workers as care providers. From the residents' perspective, accepting help may lead to loss of social power, and care practices may come to structure the rhythm of everyday life [59]. When residents interpret their roles as care recipients in relation to care workers, this may result in situations identified in this study, where a resident delegates mobility decisions to care workers, reinforcing their passive role. Furthermore, this contributes to normalizing passivity culture, where passive activities such as resting, listening to music and watching TV become a normal part of the day [2], leading to increasingly sedentary routines [7, 8]. Thus, promoting mobility may require not only resources but also critical reflection on everyday roles and assumptions of residents' capacity and agency. Care workers benefit from sufficient information about each resident's personal functional identity, habits, and history related to physical activity when supporting the maintenance of the resident's role as an active agent in their own life. In addition, the care workers awareness of how the roles are constructed in interaction is vital to avoid residents adopting a passive role.

### 4.1 Methodological Strengths and Limitations

Methodological strengths and limitations are discussed here through credibility, dependability, confirmability, and transferability [60]. The credibility of the study was strengthened by within‐method triangulation using ethnographic methods including observations and informal interviews [44]. However, some care workers' descriptions during informal interviews were brief, so this part of interpretation was partially sparse. Also, it was not possible to approach the unit without preconceptions due to researcher's dual role. Considering dependability, the researchers' influence was minimized using precise definitions of the concept (mobility) and Spradley's framework during data collection. Still, observation as a data collection method is limited since the observer can only be at one place at a time. Consistency was promoted during data collection by repeating observations with the same care worker. To enhance confirmability, reflexivity was carefully taken into consideration (see Reflexivity). During data collection, the researcher's dual role emerged as a strength as participants trusted the researcher and were willing to share their thoughts openly. Transferability was taken into consideration, and representative quotations were added to better illustrate the results. Quotations were translated from Finnish into English and adapted to plain language to ensure readability. The results represent descriptions of one ILTC unit and serve as an example for practice, training, and research to better understand mobility care in ILTC settings.

## 5 Conclusion and Implications

Mobility care can be understood from the perspective of symbolic interactionism as being constructed through social interactions between residents and care workers as well as through expected roles and assumptions embedded in the institutional care context [37, 44]. Care workers support residents' mobility in ILTC by providing person‐centred mobility care, while institutional expectations shape mobility care by influencing the need for situational compromises. In balancing these two aspects, care workers need guidance in communication with residents with dementia, sufficient resources, support, and shared practices. Mobility care should also be embedded in organizational values and guidelines to prevent mobility care from becoming a mere routine or predefined activity in a resident's day. Person‐centred and individualized mobility care can expand residents' living spaces and support their overall autonomy and decision‐making.

## Author Contributions

J.F., M.S., R.S., N.N.: design and conceptualization. J.F.: Investigation. J.F., N.N.: formal analysis. J.F., M.S., R.S., N.N.: writing – review and editing.

## Funding

This work was supported by Päivikki and Sakari Sohlberg Foundation/Päivikki ja Sakari Sohlbergin Säätiö [grant number 220066, 2022], the TYKS Foundation/TYKS Säätiö [2022], Konung Gustav V's och Drottning Victorias Stiftelse.

## Ethics Statement

This study was approved by the Ethics Committee for Human Sciences at the University of Turku on 12 December 2022 (TY/40/12.12.2022), and an amendment was made on 25 September 2023 (TY/699/06.01.01/2023) and on 12 February 2024 (TY/131/06.01.01/2024).

## Consent

A research permit was obtained from the organization (2023‐001224 T 12 02 01). All participants provided written informed consent prior to participating. The study involved participants with dementia, for whom consent to participate was provided by their family members if staff evaluated them as being unable to provide consent on their own.

## Conflicts of Interest

First author has worked part‐time in the unit under study. This relationship is described in the manuscript. The remaining authors declared no other potential conflicts of interest with respect to the research, authorship, and/or publication of this article.

## Data Availability

The data that support the findings of this study are not publicly available due to privacy and ethical restrictions.
